# An Account of a Mode of Practice Which Has Been Successfully Adopted, in Cases of Distortion of the Pelvis, in Pregnant Women

**Published:** 1800

**Authors:** John Barlow

**Affiliations:** Surgeon at Bolton in Lancashire.


XVIII. *An Account of a Mode of Practice which has been ſucceſsfully adopted, in Caſes of Diſtortion of the Pelvis, in pregnant Women. By Mr.* John Barlow, *Surgeon at Bolton in Lancaſhire. Communicated in a Letter to Mr.* W. Simmons, *Surgeon, at Mancheſter; and by him to Dr.* Simmons.

*To Mr.* Simmons, *Surgeon, at Mancheſter.*

Dear Sir,

I Have drawn up an account of the mode of practice which I have adopted, and have purſued during ſeveral years, in caſes of diſtortion of the pelvis, in pregnant women. Theſe obſervations I have thought it proper to addreſs to you, as they bear a ſtrong relation to to a fubject which you have difcuffed with great ability and fuccefs. Upon my plan of delivery, the life of the child is not endangered, while the mother fuftains no greater rifk than that incurred in every natural labour. And I will venture farther to advance, that even the ufe of the crotchet may be fuperfeded by the method which I am about to defcribe; and that the formidable obftetrical apparatus of knives, hooks and perforators may be happily banifhed in future from the furgery.

Such large promifes would meet with little credit, if they were unfupported by facts; it will appear, however, that I am delivering the refult of much experience, and not hazarding conjectures. Even a fmaller number of facts than you will find fubjoined, would have fully eftablifhed my affertions.

My method confifts in exciting premature labour early in the feventh month of pregnancy, whenever I have been confulted in time by diftorted patients. At this period of geftation, the fmaller fize of the child's head, and the greater compreffibility of its bones, render the completion of delivery eafy, without the affiftance of inftruments; fo that the mother is not expofed to any peculiar hazard by by the practice; and the child, at feven months old, has a fufficient chance of furviving the birth.

From a number of cafes which have occurred to me, I am even perfuaded, that labour might be brought on at an earlier period, if the extreme deformity of the pelvis fhould require it, without much rifk to the mother.

It is hardly neceffary to defcribe the particular method which I employ to excite labour. It is eafy for any medical man to underftand how the membranes may be ruptured, without pain or injury to the mother. If the practice were made generally known, it might be applied to very bad purpofes. I commonly give a mixture, from which the patient is taught to expect the defired refult; every thing then goes on as in natural labour.

The fafety and certainty of this method are fuch, that fome of my patients, who had formerly been delivered by the crotchet, are now in the habit of fending, to fix with me in what week labour fhall be brought on; and the event has always anfwered their expectations and mine.

I fhall now proceed to ftate fome cafes in proof of my affertions; feveral of the following ing inftances can be attefted by my medical brethren in Bolton, who have been occafionally defired to fee the patients, when I have been otherwife engaged, after the difcharge of the waters.

### CASE I.

The wife of John Smith, a woman rather advanced in life, of a delicate habit, and much deformed both in the pelvis and fpine, had been delivered fix times by the crotchet. In all thofe labours, the waters had been difcharged feveral days (in two of them, fix days) before delivery, after violent and almoft continual pains. I brought on labour early in the feventh month, June 17, 1783, and fhe was delivered on the 21ft, with common affiftance. It was a footling cafe, and the child was born dead. On the 31ft July, 1784, I ruptured the membranes in the fame woman, at the fame period of pregnancy, and difcharged the waters; fhe was delivered on the 3d of Auguft; the child was born before I arrived, excepting the head, which was brought away with common affiftance—this child was alfo dead. The width of the the pelvis, in the narroweſt part, from the ſacrum to the pubis, I judge to be one inch and a half, and in the wideſt not more than two inches.

### CASE II.

The wife of Oliver Longworth had been twice delivered by the crotchet, after the waters had been diſcharged two or three days, during which ſhe was in almoſt continual and violent labour.

Dec. 5th, 1787, I brought on labour, and ſhe was delivered on the 7th.—The child lived three hours.

Feb. 1, 1790, I again excited labour, and ſhe was delivered on the 4th.—The child was dead.

Jan. 4th, 1793, Labour was again brought on artificially, and delivery took place on the 6th. As ſhe lived in the country, the child was born before I arrived—and was dead. The pelvis of this woman was about two inches in diameter at the narroweſt part, and two inches and a half at the wideſt.—it was otherwiſe diſtorted.

CASE

### CASE III.

The wife of John Walwork had been delivered four times by the crotchet; ſhe has ſince borne ſix children by premature labour: all were alive at the time of delivery, and three of them are now living. The narroweſt part of the pelvis, in this woman, was about two inches; the wideſt two inches and a half.

Delivery took place, ſpontaneouſly, in one of theſe pregnancies, in the ſeventh month.

### CASE IV.

The wife of George Jowel had been formerly delivered of two dead children; one by the forceps, the other by the crotchet. She has ſince borne three living children by means of premature labour, one of which died ſoon after the birth; the ſecond is now alive, and four years old; the third lived ten months. The pelvis of this woman, though not ſo much diſtorted as ſome of the others, was in no part above two inches and a half wide.

### CASE V.

The wife of Peter Blakely has had ten children; the firſt ſix were ſtill born; five of whom were delivered by the crotchet. Since that time ſhe has borne four by premature labour; two of theſe were born dead, one lived an hour, the other is now four years old.

A circumſtance well worth remarking took place, reſpecting her laſt labour. Premature labour was excited three times by art, but in her laſt pregnancy it came on without any aſſiſtance. I ſhall not venture to aſſert, that the conſtitution had acquired the habit of expelling the fœtus in the ſeventh month, in conſequence of the preceding treatment; but if other inſtances of the ſame nature ſhould be obſerved, they would furniſh a ſtrong additional argument in favour of the practice which I have recommended.

This woman was ſtrong and muſcular; the pelvis was not more than two inches and a half in diameter at the wideſt part.

I preſume

I presume it may be fairly inferred, from these cases, that when the accoucheur is consulted at an early period of pregnancy, he may, by exciting labour at a given period, preserve both mother and child, under circumstances which have hitherto been supposed, by all practitioners, to require the absolute sacrifice of one life, or to be attended with great danger to both: and that in extreme cases of distortion, the mother may be saved, by having early recourse to this method, without subjecting her to the fatigue and pain occasioned by the crotchet.

I am,

Dear Sir,

Your very obedient servant,

JOHN BARLOW.

*Bolton,*

*Dec.* 16*th*, 1799.

XIX. *Observations*

